# Steinert's syndrome presenting as anal incontinence: a case report

**DOI:** 10.1186/1752-1947-5-371

**Published:** 2011-08-12

**Authors:** Fusun Erdenen, Ahmet Burak Toros, Ayse Kubat Uzum, Sirin Sacak

**Affiliations:** 1 Istanbul Education and Research Hospital, Department of Internal Medicine, Istanbul, Turkey; 2 Istanbul Education and Research Hospital, Department of Neurology, Istanbul, Turkey

## Abstract

**Introduction:**

Myotonic dystrophy (MD) or Steinert's syndrome is a rare cause of chronic diarrhea and anal incontinence. In the presence of chronic diarrhea and fecal incontinence with muscle weakness, neuromuscular disorders such as myotonic dystrophy should be considered in the differential diagnosis.

**Case Presentation:**

We present the case of a 45-year-old Turkish man with Steinert's syndrome, who was not diagnosed until the age of 45.

**Conclusions:**

In clinical practice, the persistence of diarrhea and fecal incontinence with muscle weakness should suggest that the physician perform an anal manometric study and electromyography. Neuromuscular disorders such as myotonic dystrophy should be considered in the differential diagnosis.

## Introduction

Steinert's syndrome, also called myotonic dystrophy (MD), is an autosomal dominant inherited muscular disorder which usually presents in early adulthood, is characterized by progressive muscular weakness, myotonia, frontal baldness, lenticular opacities and testicular atrophy. Multi-organ damage may include the heart, endocrine system, gastrointestinal system, respiratory system and the central nervous system. MD is diagnosed with molecular genetic techniques by demonstration of the abnormality at the 19q13-2 locus. It is the most frequent form of adult onset muscular dystrophies with an estimated prevalence of 1/20,000 [1].

Gastrointestinal involvement is frequently observed in MD. Abnormalities of smooth muscle lead to esophageal and gastrointestinal motility disorders with mega-esophagus and mega-colon, and rarely incontinence [2,3]. Some studies suggest that neurological factors can play a role in the digestive symptoms of MD patients [4,5]. Digestive symptoms may be the initial sign of the disease. In the upper digestive tract dysphagia, heartburn, regurgitation, and dyspepsia are the most common complaints while in the lower digestive tract abdominal pain, bloating, constipation and chronic diarrhea are often reported [6]. Fecal incontinence is a relatively rare symptom in MD patients.

We present the case of a 45-year-old Turkish man who presented with chronic diarrhea and fecal incontinence as the initial symptoms to our outpatient clinic and was later diagnosed with MD.

## Case Presentation

A 45-year-old Turkish man presented with diarrhea three to four times per day, without pus or blood, and fatigue for the previous six months to our outpatient Internal Medicine clinic. Diarrhea was frequently combined with fecal incontinence which had a marked negative impact on his social life. He also described muscle weakness especially in the proximal parts of his extremities that had been present for nearly five years. His walking style had changed and he was having difficulty while climbing stairs, dressing and performing routine daily activities. He had not been examined medically for these complaints before.

He had had libido loss and impotence for the previous three years for which he had been receiving sildenafil tablets on occasion. A spermiogram made three years ago, revealed azoospermia. His family history revealed that several members of his immediate family had an undiagnosed neurological disease. One of his parents had hyperthyroidism and the other suffered from cardiac arrhythmia.

Physical examination showed that his vital signs were normal, his body mass index was 22.7 kg/m^2 ^(height: 178 cm, weight: 72 kg), and depressed affect was remarkable. His thyroid gland was non-palpable. Cardiovascular and respiratory system examinations were normal. No organomegaly or rebound tenderness was detected on abdominal examination. Neurological examination revealed weakness and atrophy in the neck, face, and extremity muscles, especially involving his distal muscles, and tetraparesis, hypo-active deep tendon reflexes and steppage gait on the right side due to distal weakness. Bilateral cataract was found on ocular examination.

Laboratory findings were as follows: whole blood count was normal; serum levels of glucose: 98 mg/dl, BUN: 12, creatinin: 0.8 mg/dl, calcium: 9.1 mg/dl, albumin: 3.9 mg/dl, sodium: 138 mmol/L, potassium: 4.2 mmol/L, creatine kinase: 315 U/L, free T4: 1.25 ng/dL (0.93 to 1.7), TSH: 1,3 uIU/mL (0.27 to 4.2), PTH: 26 pg/ml, morning cortisol: 12 μg/dL, LH: 5.5 mIU/ml (1.7 to 8.6), FSH: 12 mIU/ml (1.5 to 12.4), total testosterone: 2.7 ng/ml (2.8 to 8.0), free testosterone: 6.8 pg/ml (9 to 55), sex hormone binding globulin: 80 nmol (20 to 70).

Stool examination for ova and parasites was negative. Microscopic evaluation of feces did not reveal any blood or leucocytes. No bacterial growth was observed on stool cultures. Ileocolonoscopy demonstrated no abnormality with normal-appearing mucosa. Gastroscopic evaluation revealed antral gastritis and insufficiency of cardioesophageal sphincter. On histopathological examinations of the upper and lower gastrointestinal system endoscopic biopsy samples, no pathology was detected.

Anorectal manometric study showed that internal and external sphincters were weak with reduced resting and squeezing anal pressures. Rectal sensation was within normal limits. Internal anal sphincter relaxed normally on rectal distension.

External anal sphincter electromyography (EMG) showed myopathic features and polyphasic high-amplitude motor units. However, myotonia was not observed.

His electrocardiogram demonstrated normal sinus rhythm with no signs of ischemia. Minimal mitral and tricuspid valve insufficiencies were reported on echocardiography. An X-ray of his lumbosacral vertebrae showed minimal scoliosis.

EMG showed signs of a myopathic process in the distal muscles of his extremities. Typical myotonic discharges were recorded. These results are compatible with MD.

We performed overnight polysomnography which showed sleep efficiency of 84%; REM phase comprised 6.7% of the sleep; apnea-hypopnea index (AHI) was 23.1%; desaturation index (ODI) was 21.8%, minimum SaO2 was 82%, mean saturation was 94%. We recommended noninvasive intermittent positive-pressure ventilation.

Our patient was euthyroidic. Hypotension, hyponatremia, and hyperpotassemia were not noted. Hypothalamico-pituitary-adrenal axes were intact. However, the patient had a history of libido loss and impotence for three years. Gonadal functions were compatible with hypogonadotropic hypogonadism. Further endocrine tests were performed to exclude multiple endocrine neoplasia (MEN) syndromes. Serum prolactin, calcium, parathyroid hormone, calcitonin and gastrin levels, and 24-hour urine catecholamine levels were all in normal ranges. The patient was started on intramuscular testosterone injections of 250 mg monthly. The dose was adjusted to achieve a total testosterone concentration of 3 to 4 ng/ml (immediately before injection). His gonadal symptoms improved in a few months.

The final diagnosis of MD was confirmed with genetic evaluation.

## Discussion

Myotonic muscular dystrophy, also called Steinert's syndrome is a systemic disease characterized by myotonia or difficulty in muscular relaxation, atrophy and weakness of skeletal muscle [4].

Gastrointestinal involvement is frequently observed in MD patients, and digestive complaints may be the first sign of the disease. In some patients, the impairment of gastrointestinal function develops so gradually that they adapt with little or no awareness of any disturbance; and only a thorough anamnesis may reveal possible symptoms [3]. Diarrhea, sometimes accompanied with malabsorption, steatorrhea, and crampy abdominal pain, is a frequent complaint in MD patients [6].

Diarrhea and possibly malabsorption have been attributed to reduced peristaltic activity, leading to bacterial overgrowth [7]. For the diagnosis of bacterial overgrowth, hydrogen and methane breath tests are the most important diagnostic methods [8-11]. These tests have three major drawbacks. First, breath tests themselves are not accurate in all situations. Depending on the substrate used there is the potential, in some instances, for it to be rapidly absorbed in the proximal small intestine, leading to a false-negative result and thereby reducing sensitivity. Second, there is a lack of consensus as to what threshold should be used to define a positive test. Third, the gold standard for the diagnosis of small intestinal bacterial overgrowth remains jejunal aspirate and culture (12). We did not perform a hydrogen breath test or jejunal aspirate and culture. It is well known that norfloxacin and/or cholestyramine can alleviate this symptom. Prebiotics and probiotics also exert various beneficial effects in bacterial overgrowth but studies dealing with the therapeutic use of prebiotics or probiotics in bacterial overgrowth are limited and it is, therefore, not possible to recommend them for general clinical use. Our patient was prescribed norfloxacin twice daily which helped control his the diarrhea. Afterwards, the patient had several courses of antibiotics.

Diarrhea may be accompanied with fecal incontinence such as occurred with our patient. Anal manometric studies are not frequently performed in MD patients. A few manometric studies report a decrease in both the resting and squeezing pressure, which is consistent with our data [2,13,14]. Treatment of defecation disorders involves rehabilitation, electro-anal stimulation, kinesitherapy, and biofeedback. Abercrombie JF *et al*. showed that surgical management of anal incontinence is unsatisfactory in the long term [2]. Because of this data, we did not recommend anal surgery to our patient.

Cardiac involvement of MD causes conduction defects, arrhythmias and even sudden death. No correlation was found between the severity of skeletal muscle involvement and cardiac involvement [15]. In our case, the patient had no sign of cardiac involvement.

It is well known that Steinert's syndrome is closely related to some endocrine diseases, such as diabetes mellitus, insulin resistance, thyroidal diseases, adrenal insufficiency, hypogonadotropic hypogonadism and components of MEN syndromes [16-19].

According to our physical and hormonal examination, our patient's gonadal functions were compatible with hypogonadotropic hypogonadism as a part of Steinert's syndrome. He also had bilateral cataract, which is a typical involvement of the eyes.

There is no disease-modifying therapy available for the treatment of MD. Thus, treatment is only symptomatic. The systemic (non-neuromuscular) manifestations of MD are the most treatable aspects of the disease. Recommendations regarding management are based solely on consensus and clinical experience rather than evidence from randomized controlled trials. Management includes multidisciplinary annual follow-up.

Although diarrhea and anal incontinence are common gastrointestinal complaints in MD patients, MD is a rare cause of diarrhea and anal incontinence. Thus, a careful and systemic assessment of the patients is very important.

## Conclusion

In clinical practice, the persistence of diarrhea and fecal incontinence with muscle weakness should lead the physician to perform an anal manometric study and EMG. Neuromuscular disorders such as MD should be considered in the differential diagnosis.

## Consent

Written informed consent was obtained from the patient for publication of this case report and any accompanying images. A copy of the written consent is available for review by the Editor-in-Chief of this journal.

## Competing interests

The authors declare that they have no competing interests.

## Authors' contributions

FE, ABT and SS analyzed and interpreted the patient data. AKU was a major contributor to writing the manuscript. All authors read and approved the final manuscript.
